# Agenesis of the Intrahepatic Inferior Vena Cava: A Case Report and Literature Review

**DOI:** 10.7759/cureus.35589

**Published:** 2023-02-28

**Authors:** Peter Gerges, Arooj Mian, Gurdeep Singh, Mena Aziz, Shady Guirguis, Ayman Koteish

**Affiliations:** 1 Internal Medicine, AdventHealth Orlando, Orlando, USA; 2 Hepatology, AdventHealth Orlando, Orlando, USA; 3 Gastroenterology and Hepatology, AdventHealth Orlando, Orlando, USA; 4 Medicine, The Johns Hopkins University, Baltimore, USA; 5 Medicine, University of Central Florida, Orlando, USA; 6 Medicine, Florida State University, Tallahassee, USA

**Keywords:** acute alcoholic hepatitis, hepatic vascular anomaly, deep vein thrombosis (dvt), intrahepatic ivc, inferior vena cava anomaly, agenesis of the ivc

## Abstract

Anomalies of the inferior vena cava (IVC) are an uncommon finding in the general population. A wide range of IVC anomalies has been described in the literature, the majority of which lack clinical significance. Agenesis of the IVC (AIVC) is a rare anomaly of the IVC in the general population.^ ^This anomaly may involve either complete agenesis of the IVC or agenesis of a segment of the IVC. Agenesis of the suprarenal segment is the most commonly occurring variant, while agenesis of the infrarenal and hepatic segments is less common. Here we report a case of agenesis of the intrahepatic segment of the IVC.

## Introduction

The inferior vena cava (IVC) is a large vein responsible for the venous return from the abdominal viscera, pelvis, and lower extremities. The IVC can be affected by a wide spectrum of congenital anomalies including duplication, absence, interruption, and left-sided location [1,2]. Here, we present a rare case of agenesis of the intrahepatic segment of IVC with azygos continuation. In this case report, we discuss the normal IVC anatomy, the embryological development of the IVC, types of congenital IVC anomalies and their presentations, diagnostic imaging, and dive into the latest literature on management. Through this discussion, we aim to familiarize clinicians with these rare anomalies and highlight their impact on guiding clinical decision-making.

## Case presentation

A 49-year-old female with a past medical history of an alcohol use disorder, pancreatitis, and prior clostridium difficile colitis presented with a three-day history of abdominal pain and diarrhea. Abdominal pain was localized to the right upper and right lower quadrants. The patient reported heavy alcohol intake for the past one year. She did not have any prior history of jaundice, ascites, esophageal variceal bleeding, hepatic encephalopathy, or a prior diagnosis of cirrhosis. Furthermore, she denied a family history of liver disease and the current or recent use of over-the-counter medications or herbal supplements. On admission, her vital signs were stable and a physical examination revealed right-sided abdominal tenderness.

Laboratory workup revealed the following- elevated alanine aminotransferase (ALT), aspartate aminotransferase (AST), total bilirubin, and international normalized ratio (INR), also showed low platelets with normal alkaline phosphatase and serum lipase (Table 1). A comprehensive serology to evaluate for viral, autoimmune, genetic, and metabolic diseases was negative. A complete toxicology screening was negative. A stool test to evaluate for an ongoing clostridium difficile infection was also negative.

**Table 1:** Patient Laboratory Results

Test	Patient Value		Reference Range
ALT (IU/L)		655	19 - 25
AST (IU/L)		1,482	10 - 36
Total bilirubin (mg/dL)		2.6	0.1 - 1.2
Alkaline phosphatase (U/L)		123	44 - 147
Serum albumin (g/dL)		3.5	3.5 - 5.5
INR		1.28	0.8 - 1.1
Platelets (/µL)		59,000	150,000 - 450,000
Serum lipase (U/L)		37	0 - 160

The initial working diagnosis included acute alcoholic hepatitis. However, the pattern of elevated liver enzymes, specifically the sharp rise in aspartate aminotransferase (AST), could not be explained by alcoholic injury alone. In order to assess for other contributing factors, such as hepatic vascular compromise, abdominal imaging was performed. A triple-phase computed tomography (CT) of the abdomen and pelvis with intravenous (IV) contrast revealed significant abnormalities of the entire intra-abdominal vasculature.

**Figure 1:**
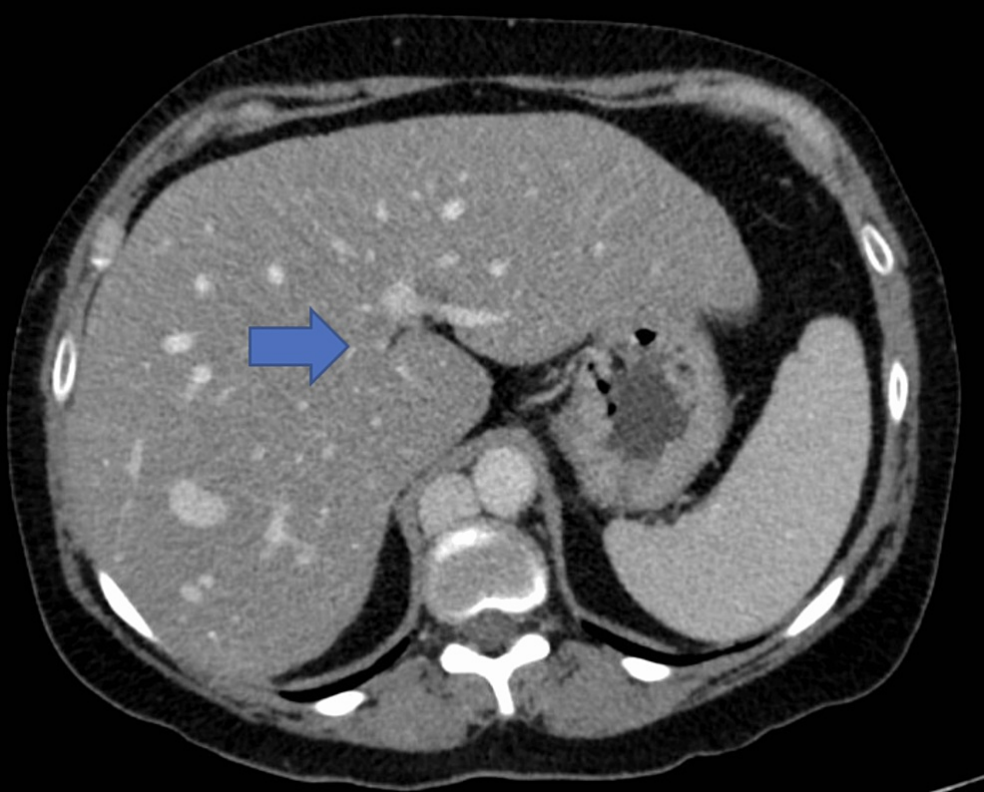
CT abdomen and pelvis, axial reconstruction, reveals the complete absence of the intrahepatic part of the inferior vena cava (blue arrow)

**Figure 2:**
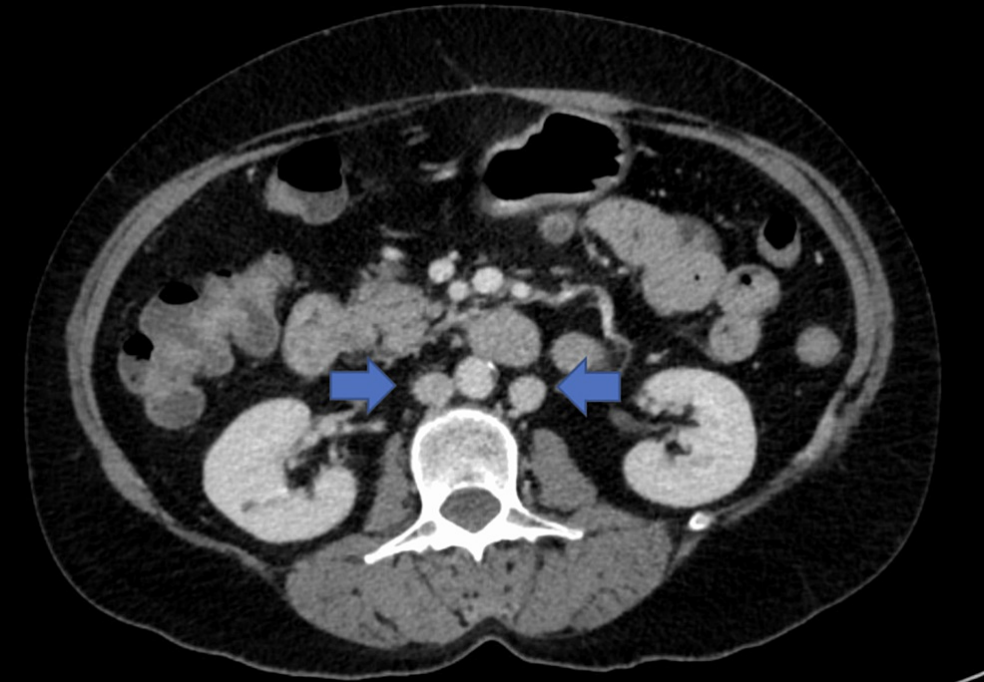
CT abdomen and pelvis, axial reconstruction, shows duplicated infrarenal inferior vena cava (blue arrows)

**Figure 3:**
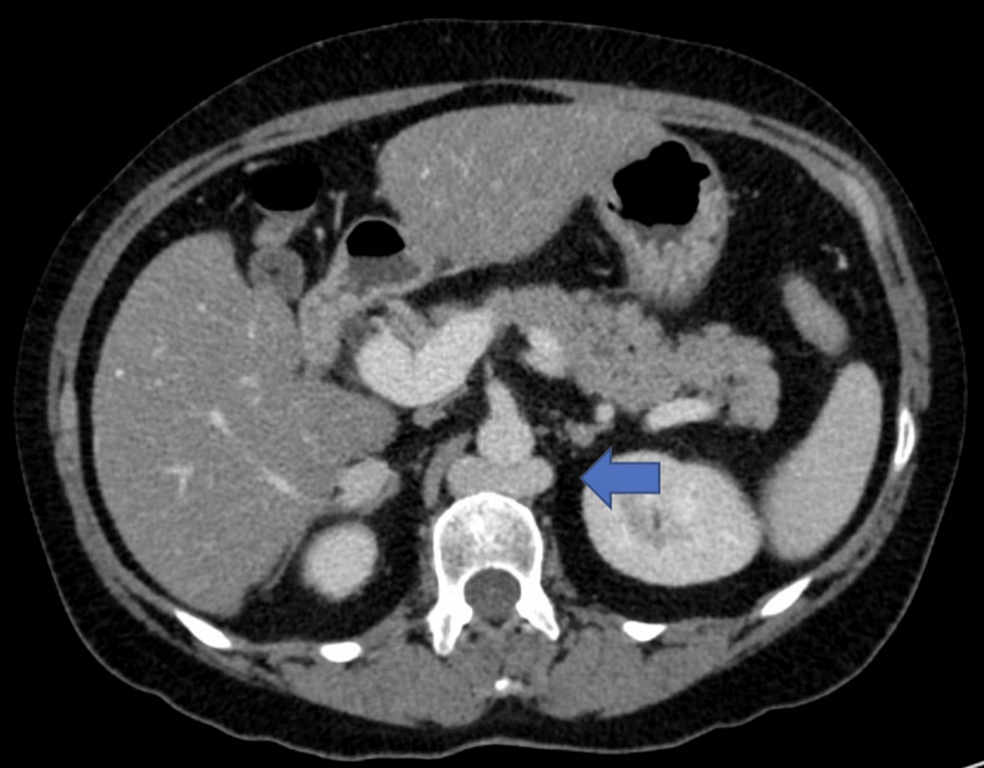
CT abdomen and pelvis, axial reconstruction, shows left renal vein draining into the dilated azygous system (blue arrow)

**Figure 4:**
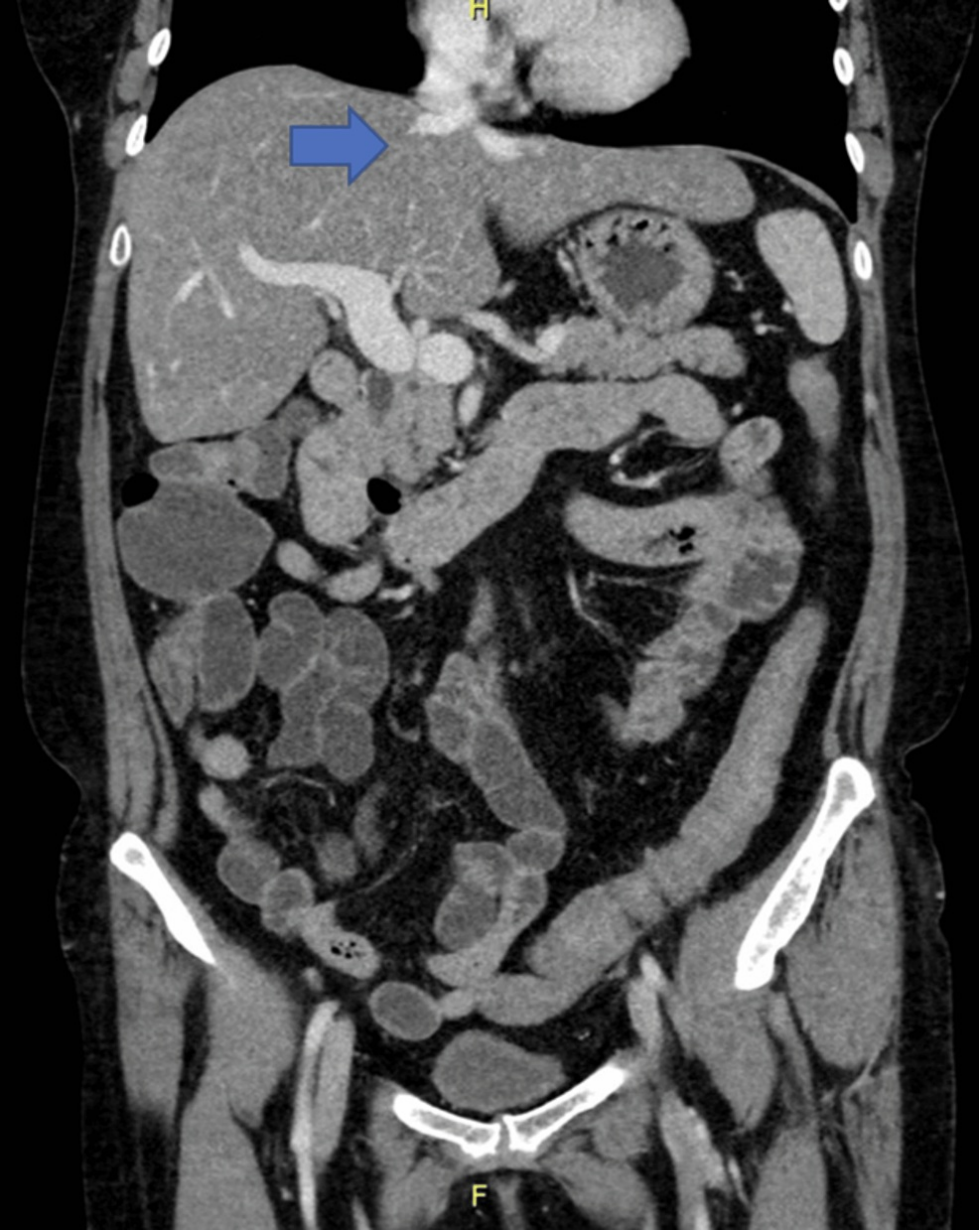
CT abdomen and pelvis, coronal reconstruction, shows the diminutive right and left hepatic veins draining into the suprahepatic inferior vena cava remnant (blue arrow)

Specifically, there was an absence of the intrahepatic segment of the IVC (Figure 1), duplicated infrarenal IVCs with azygous continuation (Figure 2), a spontaneous intrahepatic shunt from the right renal vein to the right hepatic vein coursing through the right hepatic lobe, the left renal vein draining directly into the dilated azygous system (Figure 3), and an abnormal outflow of the liver involving diminutive right and left hepatic veins draining directly into the suprahepatic IVC remnant (Figure 4). The portal and hepatic veins were patents. There was no evidence of pancreatitis, however diffuse hepatic steatosis was noted. Further imaging with CT angiography and CT venography of the chest, abdomen, and pelvis confirmed the interrupted IVC with azygous continuations. Otherwise, no intrathoracic vascular anomaly or congenital variations were found. A liver biopsy was not performed due to the complex vascular anatomy and the minimal impact it would have on guiding management.

Given the aforementioned findings, the patient's presentation was attributed to an episode of acute alcoholic hepatitis with an exaggerated response of liver enzyme elevation in the setting of an underlying abnormal hepatic vasculature. It was postulated that alcoholic liver injury was compounded by hypovolemia of her diarrheal illness, and potentially exacerbated by the underlying congestive hepatopathy related to the hepatic IVC anomaly. This process led to a higher-than-expected rise in liver enzymes.

The patient was managed with supportive care including intravenous fluid hydration. Her symptoms resolved and the liver enzymes gradually normalized over the hospital course. She was discharged in stable condition and advised to follow up with hepatology in the outpatient setting.

## Discussion

The IVC is the largest vein in the body. It drains blood from the lower extremities, pelvis, and abdominal viscera into the heart. It is formed by the confluence of the right and left common iliac veins at the level of the fifth lumbar vertebrae. It then ascends through the abdomen in close proximity to the abdominal aorta, passes behind the liver, and courses through the diaphragm to enter the thoracic cavity, terminating at the right atrium.

**Figure 5:**
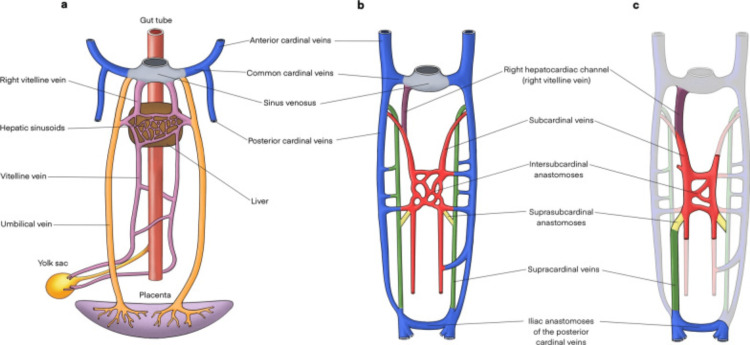
Embryological development of the IVC Licensed under Creative Commons Attribution 4.0 International License [1].

**Figure 6:**
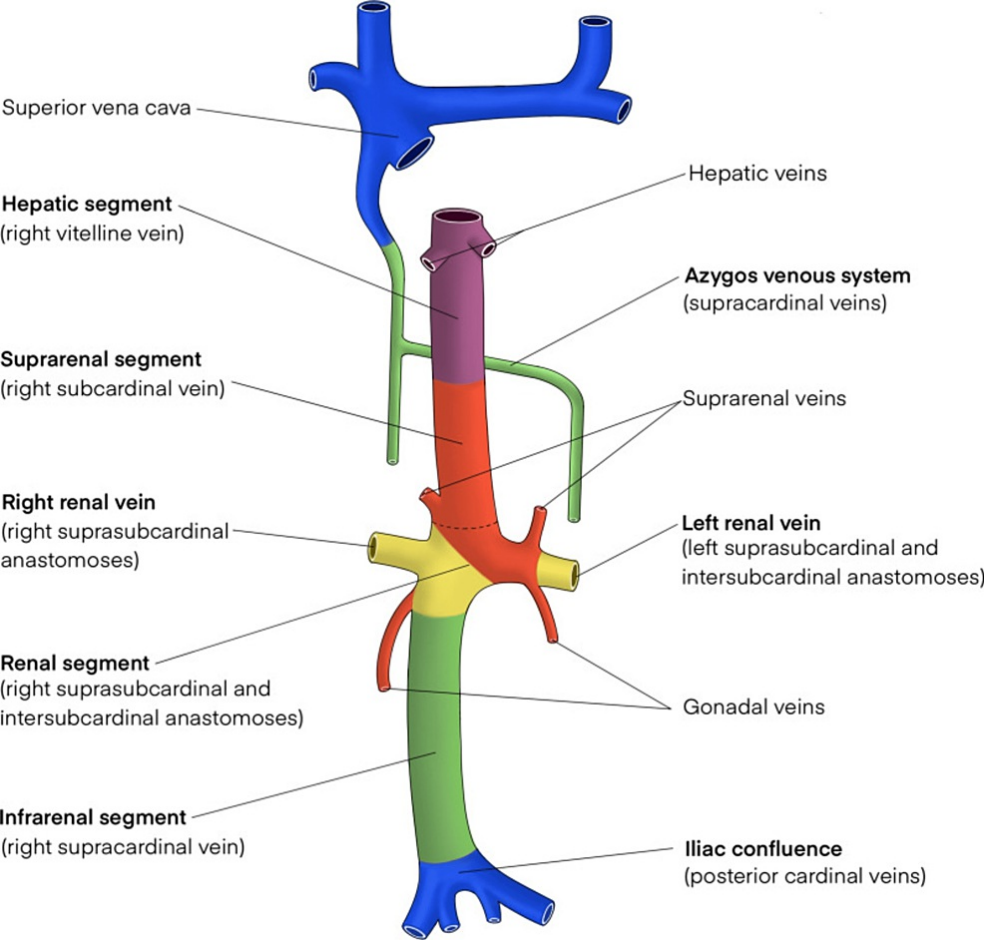
The mature IVC is composed of four segments: hepatic, suprarenal, renal and infrarenal segments Licensed under Creative Commons Attribution 4.0 International License [1].

The IVC develops from a complex embryological process between the sixth and eighth weeks of gestation. It arises from three pairs of primitive veins: the posterior cardinal veins, the subcardinal veins, and the supracardinal veins, which appear in this order (Figure 5) [1,3]. Subsequently, some of these embryological veins regress while others anastomose to ultimately give rise to the IVC. The mature IVC is composed of four segments- the hepatic, suprarenal, renal, and infrarenal segments (Figure 6) [1].

Congenital anomalies of the IVC result from abnormal persistence or regression of these embryological veins and may include left-sided IVC, duplicate IVC, and agenesis of the IVC [1]. The underlying causes leading to the developmental failure of IVC are not fully understood. Nonetheless, it has been hypothesized that embryonic dysontogenesis and intrauterine or perinatal thrombosis may play a role [4-7].

Agenesis, or absence, of the IVC, is one of the most uncommon anomalies of the IVC with a prevalence of 0.0005%-1% in the general population as described above [8]. AIVC can be further characterized by the segment involved. The three commonly described variants of AIVC include the absence of the suprarenal IVC, the absence of the infrarenal IVC, and the absence of the entire IVC [9]. We present a case of absence of the intrahepatic IVC with azygous continuation. It is a rarely described anomaly and has been reported only a few times in literature. In the case presented, the following anatomical configurations were seen: (1) the absence of the intrahepatic IVC; (2) duplicated infrarenal IVCs draining into a dilated azygos vein; (3) the left renal vein draining directly into the dilated azygos vein; (4) the right renal vein draining into the right hepatic vein via a spontaneous shunt coursing through the right hepatic lobe; (4) the suprarenal IVC bypassing the interrupted segment and draining into the azygous/hemiazygos venous system; and (5) the diminutive right and left hepatic veins draining directly into the suprahepatic IVC remnant.

IVC anomalies lead to the development of extensive collateral circuits in the abdomen and lower extremities which bypass the interrupted segment to allow delivery of venous blood to the right atrium, as seen in our case. These collateral venous networks commonly involve the azygous vein, hemiazygos vein, lumbar vein, paravertebral veins, and anterior abdominal wall veins. These veins often become extensively dilated and may be mistaken for adenopathy or venous occlusion by a neoplasm [10]. Hence, knowledge of these variations may aid in establishing an accurate diagnosis. Furthermore, these anatomical malformations may lead to difficulties in vascular access and placement of IVC filters [10]. Hence, a description of these anomalies and their collateralization systems by radiologists may help guide clinicians while managing such patients.

AIVC may be an asymptomatic incidental finding or present with vague abdominal or lower back pain. More commonly, however, AIVC presents with a deep vein thrombosis (DVT) of the lower extremities or pelvic region, especially in young patients. These vascular anomalies promote venous blood stasis and endothelial damage, serving as a risk factor for the development of DVTs [7]. Moreover, patients may also present with chronic venous insufficiency, venous ulcerations, and stasis dermatitis [5,11].

IVC anomalies can be diagnostically challenging. Nevertheless, imaging modalities such as CT angiography (CTA), magnetic resonance imaging (MRI), and venography can provide detailed descriptions of the IVC and are considered the best imaging techniques to identify these anomalies [10]. However, for patients presenting with a DVT, duplex vascular ultrasound is usually performed as the initial imaging test. Subsequently, a CTA with MRI or venography is recommended for all patients with an idiopathic DVT above the inguinal ligament [12]. Despite these imaging techniques, IVC anomalies can be difficult to detect and may be mistaken for neoplasms or adenopathy as mentioned above [10]. Due to these factors, it is important for radiologists to familiarize themselves with the wide spectrum of IVC variants as recognition and knowledge of these entities are essential for guiding clinical decision-making.

 As discussed above, IVC anomalies may serve as a possible risk factor for the development of deep vein thrombosis and chronic venous insufficiency. As such, the detection of these anomalies is essential as they carry significant implications in patient management.

However, due to the rarity of these entities, medical literature remains sparse and the management of patients with AIVC is not well established. On review of the literature, patients who presented with AIVC and DVT were often treated conservatively with anticoagulation therapy [4,13,14]. However, the type of anticoagulant agent and the optimal duration of therapy remains unclear. Available studies advocated for long-term or indefinite anticoagulation due to the potentially higher risk of DVT recurrence in patients who discontinued anticoagulation [5,6,15]. In one study performed by Oblitas et al., nine patients with AIVC/DVT were started on long-term anticoagulation therapy. At a follow-up nearly 78 months later, two patients who were no longer on anticoagulation therapy had a recurrence of DVT while seven patients who remained on anticoagulation did not [16]. Similar data was seen in other case reports [17,18].

Alternatively, thrombolytic therapy may also be considered for the treatment of patients with AIVC and DVT. There are five case reports of AIVC/DVT treated by catheter-directed thrombolysis; of these, only three were treated successfully [17]. While catheter-directed thrombolysis has been associated with rapid relief of symptoms and reduction of thrombus burden, this approach may be challenging due to the difficult vascular anatomy [19]. Furthermore, performing thrombolytic therapy in this chronically obstructed vascular condition may increase the risk of DVT recurrence [20]. Open surgical reconstruction may be considered a possible therapeutic approach in patients with chronic venous insufficiency [21].

## Conclusions

Agenesis of the IVC is an extremely rare congenital vascular anomaly. We recommend prophylactic anticoagulation as an individualized medical decision. The benefits of initiating prophylactic anticoagulation to reduce the risk of DVTs in a patient with AIVC should be weighed against the patient’s risk of hemorrhagic events.

Furthermore, all patients with AIVC should be educated about the increased risk of DVTs associated with their underlying anomalies. These patients should be advised to avoid additional DVT risk factors including prolonged immobilization, oral contraceptive use, anabolic steroid use, and herbal supplement use. To prevent venous insufficiency, we recommended leg elevation and compression stockings. Finally, an effort should be made to contact the primary care providers of these patients to inform them about their patients' anomalies and the associated risk of DVTs.
